# Combined petrosal approach: a systematic review and meta-analysis of surgical complications

**DOI:** 10.1007/s10143-023-02072-7

**Published:** 2023-07-13

**Authors:** L. Giammattei, D. Starnoni, D. Peters, M. George, M. Messerer, R. T. Daniel

**Affiliations:** 1 Department of Neurosurgery, Lausanne University Hospital, Lausanne, Switzerland; 2 Faculty of Biology and Medicine, University of Lausanne, Lausanne, Switzerland; 3 Carolina Neurosurgery & Spine Associates, Charlotte, NC USA; 4 Department of Otorhinolaryngology and Head and Neck Surgery, Lausanne University Hospital, Lausanne, Switzerland

**Keywords:** Combined petrosal approach, Combined transpetrosal approach, Presigmoid approach, Retrolabyrinthine approach, Petrosal approaches

## Abstract

Transpetrosal approaches are known to be associated with a significant risk of complications, including CSF leak, facial palsy, hearing impairment, venous injury, and/or temporal lobe injury. We aimed to evaluate the morbidity of the standard combined petrosal approach (CPA), defined as a combination of the posterior (retrolabyrinthine) and the anterior petrosal approach. We performed a systematic review and meta-analysis of articles reporting on clinical series of patients operated on for petroclival meningiomas through CPA. Studies that used the terminology “combined petrosal approach” without matching the aforementioned definition were excluded as well as clinical series that included less than 5 patients. A total of 8 studies were included involving 160 patients. The pooled complication rates were 3% (95% CI, 0.5–5.6) for CSF leak, 8.6% (95% CI, 4.1–13.2%) for facial palsy, 8.2% (95% CI, 3.9–12.6%) for hearing impairment, 2.8% (95% CI, 0.9–6.5%) for venous complications, and finally 4.8% (95%, 1.2–8.4%) for temporal lobe injury. Contrary to the general belief, CPA is associated with an acceptable rate of complications, especially when compared to alternative approaches to the petroclival area. In view of the major advantages like shorter trajectory, multiple angles of surgical attack, and early tumor devascularization, CPA remains an important tool in the armamentarium of the skull base surgeon.

## Introduction

The terminology for transpetrosal approaches (TPAs) is confusing and often ambiguous [16]. In particular, the definition of “combined petrosal approach” is variable among published studies. The standard definition is the combination of an anterior and posterior petrosectomy with preservation of the semicircular canals [17]. However, it is common for alternatives with variable amounts of petrosal drilling to be labeled as combined petrosal approaches. Consequently, the rate of morbidity of the standard approach is unclear, as multiple different approaches with significant variations are grouped under the umbrella term of combined petrosal approach (CPA) [28].

With this paper, we aimed to investigate the morbidity of CPA, defined as a combination of the posterior (retrolabyrinthine) and anterior petrosal approach [5] in published series of petroclival meningiomas (PCMs). We performed a literature review focusing on the most common complications associated with CPA including cerebrospinal fluid (CSF) leak, facial palsy, hearing impairment, venous injury, and temporal lobe injury. The results are discussed and compared to commonly used alternative approaches.

## Methods

### Search strategy and selection criteria

Following PRISMA (Preferred Reporting Items for Systematic Reviews and Meta-Analyses) guidelines and recommendations, we conducted a systematic search using PubMed. All studies were screened without a backward date limit. The following medical subject headings (MeSH) and free text term were used: “combined petrosal approach” OR “combined transpetrosal approach” OR “retrolabyrinthine approach” OR “presigmoid approach.” The related articles function was used to find additional studies. Only articles in English were considered. We manually reviewed the reference list of identified studies for further inclusions.

### Study selection

Two authors (L. G. and D. S.) independently reviewed the titles and abstracts to assess eligibility. Inclusion criteria were (1) surgical series of PCMs treated through CPA defined as a combination of the posterior (retrolabyrinthine) with the anterior petrosal approach and (2) series including at least 5 patients. Studies that used the terminology “combined petrosal approach” without matching these two criteria were excluded. Studies that did not stratify complications according to the employed surgical approach were excluded.

### Data extraction

The title and abstract of each study were screened for relevance by 1 investigator (L. G.). Full-text articles were reviewed against specific inclusion criteria. Two authors (L. G. and D. S.) independently extracted data focusing on the rate of CSF leak, facial palsy, hearing impairment, venous injury (including sinus thrombosis and venous infarction), and temporal lobe injury (swelling, edema, contusions, hemorrhages). Other reported complications were also noted.

### Statistical analysis

Categorical variables were expressed as number and percentage. Quantitative variables were expressed as means with minimum and maximum. Weighted summary rates were determined using meta- analysis models. Testing for heterogeneity was performed for each meta-analysis. In case of heterogeneity, binary random-effects model (DerSimonian**–**Laird method) was used in some of the analysis assuming that the included studies were a random sample from a hypothetical population of CPA operated on for a PCM; otherwise, a binary-fixed effect model with inverse variance weighting was employed. When 0% complication rates were reported, variances were estimated as pooled variances obtained from the other studies. The OpenMeta (Analyst) from the Agency for Healthcare Research and Quality was used to perform these analyses. Pooled estimates using meta-analytical techniques were obtained for CSF leak, facial palsy, hearing impairment, venous injury, and temporal lobe injury.

## Results

### Study selection

**Fig. 1:**
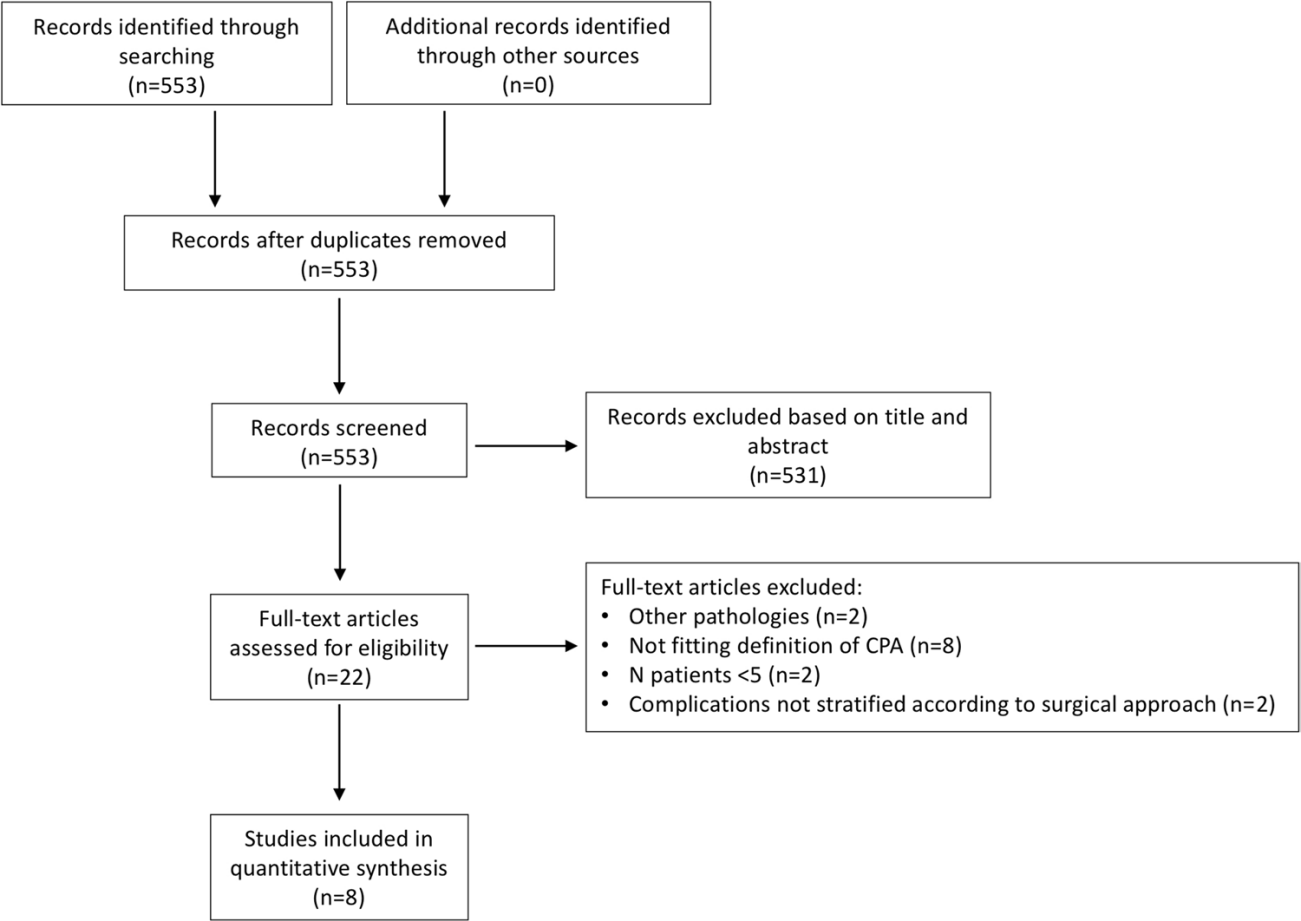
Flowchart of the study selection process

The literature search identified 553 articles, which were then screened based on the title and abstract. Among them, 22 articles were retrieved for full-text analysis. After careful evaluation, 8 articles met inclusion criteria. The flowchart of article selection is shown in Fig. 1.

### Demographics and clinical data

One hundred sixty patients operated on for a PCM were included in the meta-analysis. Female to male ratio was 3.5:1. Mean age was 52 years old. Two out of 126 (1.6%) of patients had been previously operated. Mean follow-up was 40 months. GTR was obtained in 67% of cases. Recurrence was described in 4.3% of patients. The complete population characteristics are represented in Table 1.

**Table 1:** Population characteristics

Reference	*N*	Pathology	GTR, NTR (%)	M:F	Mean age	Previously operated tumor	Follow-up (months)	Recurrency	Average tumor size
Cho and Al-Mefty (2002) [5]	7	Petroclival meningiomas	5 GTR, 2 NTR	1:6	42 (19–56)	1/6	24 (2–60)	0	36 × 35 × 42 mm
Erkmen et al. (2005) [9]	34	Petroclival meningiomas	NR	NR	NR	NR	NR	NR	NR
Baugh et al. (2007) [3]	19	Petroclival meningiomas	17 GTR (89%)	3:16	51 (24–67)	1/19 (5%)	22.8	0	31 mm
Kusumi et al. (2012) [20]	23	Petroclival meningiomas	11 GTR (47.8%), 7 NTR (30.4%)	5:18	48 (29–66)	0	NR	NR	95.7% > 40 mm
Shibao et al. (2015) [37]	8	Petroclival meningiomas	3 GTR (37.5%), 3 NTR (37.5%)	3: 5	53.8 (43–71)	0	30 (1–81.6)	0	43.1 (22–66) mm
Morisako et al. (2015) [26]	36	Petroclival meningiomas	92.7% (48–100%) (mean EOR)	NR	54.9	0	77.9 (7–157)	2/36 (5.5%)	32.1 cm^3^ (mean volume)
Morisako et al. (2021) [27]	23	Petroclival meningiomas	17/23 (74%) EOR >96%	4:19	54 (37–74	0	29.4 (4–74)	2/23 (86.9%)	40.3 (30–74)
Piper et al. (2023) [28]	10	Petroclival meningiomas	2 GTR (20%) 2 NTR (20%)	4:6	57.8 (34–72)	0	58.8 (12–192)	0	43 × 36 mm

*NR* not reported, *GTR* gross total resection, *NTR* near total resection, *EOR* extent of resection

### Complications

**Fig. 2:**
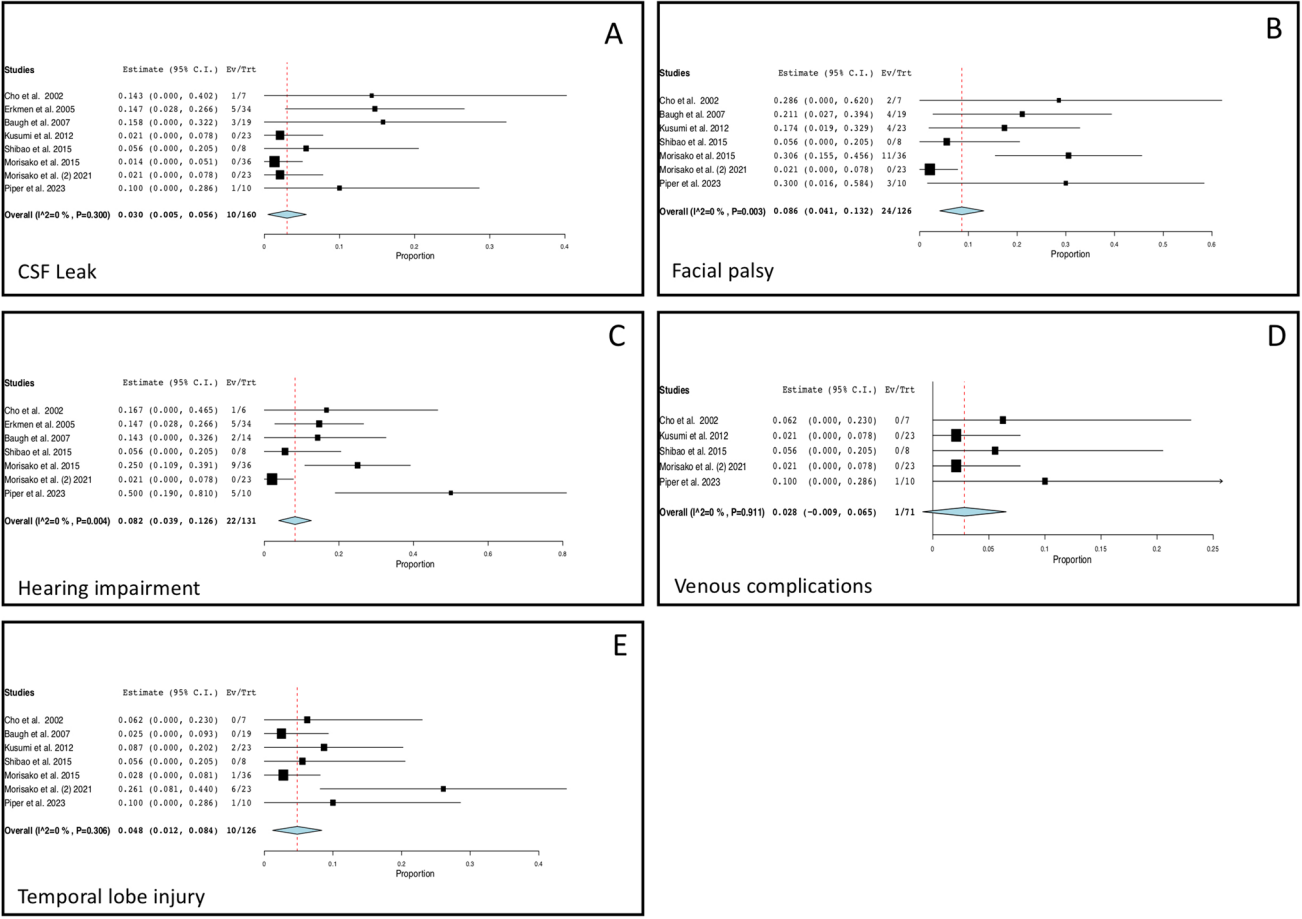
Pooled rate of CSF leak (**A**), facial palsy (**B**), hearing impairment (**C**), venous complications (**D**), and temporal lobe injury (**E**). Ev/Trt, number of events and number treated

We used meta-analytical techniques to obtain pooled estimates of surgical complications (Fig. 2). The raw data are presented in Table 2. Tests for heterogeneity were *p* = 0.3 for CSF leak, *p* = 0.003 for facial palsy, *p* = 0.004 for hearing impairment, *p* = 0.911 for venous complications, and *p* = 0.306 for temporal lobe injury. The pooled complication rates were 3% (95% CI, 0.5–5.6) for CSF leak, 8.6% (95% CI, 4.1–13.2%) for facial palsy, 8.2% (95% CI, 3.9–12.6%) for hearing impairment, 2.8% (95% CI, 0.9–6.5%) for venous complications, and 4.8% (95%, 1.2–8.4%) for temporal lobe injury. Issues related to trigeminal nerve were described in 5/160 patients (3.1%).

**Table 2:** Clinical outcome and complications

Reference	*N*	CSF leak (%)	CNVII palsy (%)	CNV issues (%)	Hearing impairment (%)	Venous complications (%)	Temporal lobe injury (%)	Stroke	Other complications (%)
Cho and Al-Mefty (2002) [5]	7	1 (14)	2 (28)	3 (2 dry-eye, 1 keratitis) (42)	1/6(16)	0	0	0	1 (14) Pneumonia
Erkmen et al. (2005) [9]	34	5 (15)	NR	0	5 (14.7)	NR	NR	NR	0
Baugh et al. (2007) [3]	19	3 (19)	4 (21)	1 neuralgia(5.3)	2/14 (14)	NR	0	0	1 Abdominal hematoma 1 post-op seizure
Kusumi et al. (2012) [20]	23	0	4 (17)	0	NR	0	2 (8)	0	0
Shibao et al. (2015) [37]	8	0	0	0	0	0	0	0	0
Morisako et al. (2015) [26]	36	0	11 (30.6)	0	9 (25)	NR	1 (2.8)	0	0
Morisako et al. (2021) [27]	23	0	0	0	0	0	6 (26)	0	0
Piper et al. (2023) [28]	10	1 (10)	3 (33)	1 keratitis (10)	5 (50)	1 (10)	1 (10)	4	1 (10) wound dehiscence

## Discussion

TPAs have been conceived to address lesions of the petroclival region, such as PCMs. The rationale for these complex approaches is to shorten the distance to the lesion, widen the surgical exposure, and provide early devascularization. However, the literature shows a progressive tendency to abandon them in favor of traditional approaches that do not require petrous bone drilling, such as the retrosigmoid approach (RSA) in combination with a supratentorial craniotomy [33]. This trend towards alternative strategies comes mainly from the fact that TPAs are believed to be associated with high risk of complications, including CSF leak, facial palsy, hearing impairment, venous injury, and temporal lobe injury, in addition to the fact that they are extremely time consuming.

The term combined petrosal approach is sometimes associated with the use of a posterior petrosectomy (retrolabyrinthine, translabyrinthine, or transcochlear) combined with a temporal and retrosigmoid craniotomy [2, 6, 23, 36], generating confusion and heterogeneity in the interpretation of the results presented in the literature. This misunderstanding is also reflected by a review of the literature that included surgical series with different definitions of CPA [17]. As a consequence the rate of morbidity remains unclear for the standard CPA [28].

The CPA evolves from the description of Hakuba, who first described an anterior petrosectomy combined with a posterior petrosectomy, including removal of posterior and superior semicircular canal and medial part of the horizontal semicircular canal [15]. Sekhar et al. [34] subsequently developed the concept of partial labyrinthectomy and petrous apicectomy, where the petrous apex is partially removed though the corridor created by the labyrinthectomy. A further anatomical study [4] pointed out that the advantage in terms of exposure was mostly provided by the petrous apicectomy rather than the partial labyrinthectomy. This understanding has progressively led to the development of the standard CPA defined as the combination of the anterior and posterior petrosal retrolabyrinthine approach, balancing approach-related morbidity with improved exposure of the petroclival area [5].

The main indication for TPAs is represented by PCMs. Surgery for these tumors is associated with significant cranial neuropathies and complications irrespective of the approach used [7]. In addition, when alternative approaches such as RSA are chosen, more than one surgery could be required to access the entirety of the tumor. The necessity of adjuvant treatment (radiosurgery or radiotherapy) is likely to be higher when these alternative approaches are used. Therefore, the morbidity of these procedures has to factor in the combined morbidity of multiple treatment modalities. This meta-analysis attempted to evaluate the rate of complications frequently attributed to the CPA.

### CSF leak

Pooled overall rate of CSF leak was 3%. This data appears to be in line with alternative approaches to the petroclival region. The Montano et al. series of 76 patients operated through RSA for tumors of the cerebello-pontine angle experienced a 15.5% rate of CSF leak [25]. However 60% of the patients included in this series were represented by vestibular schwannomas (VS). The high rate of CSF leak may be explained by the opening of the internal acoustic canal (IAC) that is usually required during VS surgery, rather than by the RSA itself. A recent study describing complications of the RSA in a large series of 449 oncological cases described a rate of CSF leak of 7.6% [1]. Again, this study included a relatively high number (40%) of VS that may have required IAC drilling, which could explain why these rates of CSF leak are higher than our reported rate of 3%. For PCMs, which represent the pathology included in our review, large surgical series employing standard craniotomies (such as retrosigmoid, pterional, subtemporal, or a combination between these techniques) described a rate of CSF leak ranging from 3 to 5% [32, 41]. A series of 32 patients harboring a PCM where a purely endonasal endoscopic approach was used reported a CSF leak rate of 28.1% [19]. A recent series of various tumors involving the petroux apex treated with endoscopic endonasal or transorbital approach reported a 10% rate of CSF leak [21].

### Facial nerve morbidity

Our meta-analysis found a rate of post-operative facial palsy of 7.1%. All the patients included in our analysis were treated for a PCM, a complex pathology with a known postoperative morbidity [12, 33]. Large surgical series of PCMs resected through RSA found a rate of facial nerve dysfunction between 8 and 23% [32, 41]. A systematic review and meta-analysis of the literature focused on the risk of post-operative cranial nerve deficit after surgery for PCM found a rate of facial palsy of 11% for combined petrosal, 23.5% for retrosigmoid intradural suprameatal approach, and 5.6% for standard RSA [7]. The risk of facial nerve palsy for PCMs is significant regardless of approach chosen, and the CPA does not seem to increase this risk. On the contrary, CPA avoids operating through a small window between the trigeminal and facial nerve such as with RSA.

### Hearing impairment

Our meta-analysis showed a rate of post-operative hearing impairment of 7.7%. This data appears quite similar to the findings emerging from large series of PCMs treated with traditional craniotomies that described an 11% rate [32]. Similar rates have also been described by other authors employing RSA for posterior fossa meningiomas [14, 40, 42]. Di Carlo et al. [7] in their meta-analysis found a rate of post-operative hearing loss of 11.6% for the retrosigmoid intradural suprameatal approach and 14.8% for the combined petrosal approach. Fatima et al. [10] in their series of posterior fossa meningiomas revealed that patients undergoing surgery through RSA had significantly higher risk of a poor hearing outcome compared to a petrosal approach. These authors concluded that TPAs are therefore indicated for supra- or pre-meatal tumors with internal acoustic canal involvement in patients with good hearing. They also found TPAs indicated in most patients without useful hearing, thanks to the advantages provided in terms of wider operative field, superior illumination, ability to de-vascularize the tumor, and improved working access around cranial nerves.

### Venous issues

Pooled overall rate of venous injury was 2.8%. The first venous structure at risk during a CPA is the sigmoid sinus. Jean et al. [18] focused on venous sinus compromise for 52 patients operated through presigmoid approach. Five patients (10%) developed a narrowed sigmoid sinus, whereas 5 patients (10%) were diagnosed with asymptomatic sinus thrombosis. None of these patients experienced symptoms. Shabo et al. [35] also investigated the incidence of asymptomatic sinus thrombosis after posterior fossa tumor surgery and found a rate of 12.4%. Considering that unilateral sigmoid sinus constriction or thrombosis is usually asymptomatic, this complication is frequently underdiagnosed and not reported in most of the surgical series.

TPAs also put the vein of Labbé at risk, especially if it has a long, low horizontal course along the temporal lobe. This anatomical configuration increases the risk of venous injury during the craniotomy and with temporal lobe retraction, reducing tolerance for stretching of the vein [8]. Injuries to vein of Labbé during RSA are not reported; this is often cited as a clear advantage over CPA.

Great care should be also taken with petrosal bridging veins, and meticulous preoperative analysis of individual venous anatomy is mandatory [31]. When performing the CPA, the surgeon should be aware of the sphenobasal or sphenopetrosal routes of drainage that may be interrupted to expose the petrous apex [24]. Drainages route of the superficial middle cerebral vein should be preserved. An anterior entrance of the superior petrosal vein complex into the superior petrosal sinus should be detected because this can limit the posterior tentorial cutting[8].

### Temporal lobe injury

Our meta-analysis showed a rate of temporal lobe injury of 4.8% (10/126). Six out of 10 patients come from the series of Morisako et al. [27]. These authors specified that only one of their patients developed a small hemorrhage, while the others showed asymptomatic temporal lobe edema not related to venous injury. Part of the benefit of TPAs is a reduction in brain retraction, but no approach can eliminate it completely. Studies focused on the extradural anterior petrosal approach have previously demonstrated that the morbidity for the temporal lobe is between the 3 and 6% [11, 39]. The rate of 4.5% compares favorably to the results of the purely intradural subtemporal approach [30, 38]. The subtemporal approach, often used with a tentorial incision to increase the surgical exposure, has a significant risk of retraction injury of the temporal lobe parenchyma and vein of Labbé [30, 38]. In particular, Smith et al. [38] reported a 12.5% incidence of postoperative aphasia due to temporal lobe swelling in a series of patients with cavernous malformations who were all treated by a subtemporal approach. Sabatino et al. [30] treated 10 patients using the intradural subtemporal approach and observed one patient (10%) that presented confusion, aphasia, and seizures related to temporal lobe swelling. Tentorial peeling during CPA is an emerging technique that has not yet been evaluated in a large series, but has the potential to reduce iatrogenic contusions to the temporal lobe and avoid stretching the vein of Labbé [13].

### Other complications

Other rare post-operative complications have been reported including trigeminal neuralgia in 1/160 patients (0.6%), dry eye in 2/160 (1.2%), keratitis in 2/160 (1.2%), and abdominal hematoma due to fat graft in one patient 1/160 (0.6%). Piper et al. [28] described operative times ranging between 8 and 13 h. The reported rate of wound dehiscence appears quite low 1/160 (0.6%), and no deep vein thrombosis or pulmonary embolism were described. This is in line with the experience of Raghavan et al. [29], who demonstrated that increased operative duration was not associated with postoperative complications. Their findings may alleviate concerns about long duration of surgery. Stroke with related hemiparesis has been described in the 2.5% (4/160) of the patients. This percentage is similar to the results of other recent publications about PCMs mainly treated through RSA[22, 32]. The CPA offers better control over the interface between the tumor and the brainstem as well as basilar perforators, thus possibly reducing the risk of stroke. However this complication depends on the consistency of the tumor and most of all, the intraoperative decision-making of the surgeon that should judge intraoperatively the opportunity of radical resection. After performing a long and complex approach such as the CPA, surgeons may be reticent lo leave residual tumor behind. However, the temptation for an overaggressiver radical tumor resection that puts the patient at undue risk of stroke must be avoided. [33]

### Limitations

This meta-analysis has the following shortcomings: (1) the observational nature of the available studies; (2) the small sample size of the population; (3) the same surgical group accounted for 2 studies among the 8 included; this may have affected the results; (4) the surgical series represented the results of well recognized international experts in petrosal approaches, due to which their results cannot obviously be generalized; (5) some complications like facial palsy and hearing impairment can have significant causative overlap between approach and localization-related morbidity making it difficult to determine specifically the neurological morbidity caused by CPA. Data emerging from this meta-analysis are not intended to claim the superiority of CPA over RSA. Each of these approaches have specific advantages and disadvantages, and both routes should be considered when dealing with pathologies of the petroclival region. Of note, RSA is simpler and faster to perform, likely explaining its increasing popularity in recent years.

## Conclusion

Our meta-analysis revealed a low rate of surgical complications, quite comparable to that of alternative approaches. Overall, the low morbidity rate of CPA could help demystify this procedure, especially in view of the major advantages provided by this approach. CPA is therefore a safe and useful tool in the armamentarium of the skull base surgeon.

## Data Availability

Data are available upon request.
